# Camrelizumab plus famitinib in previously chemo-immunotherapy treated patients with advanced NSCLC: results from an open-label multicenter phase 2 basket study

**DOI:** 10.1007/s00262-024-03715-4

**Published:** 2024-05-10

**Authors:** Shengxiang Ren, Anwen Xiong, Jia Yu, Xicheng Wang, Baohui Han, Yueyin Pan, Jun Zhao, Yufeng Cheng, Sheng Hu, Tianshu Liu, Yalun Li, Ying Cheng, Jifeng Feng, Shanyong Yi, Shanzhi Gu, Shegan Gao, Yongzhong Luo, Ying Liu, Caigang Liu, Huijie Duan, Shuni Wang, Xinfeng Yang, Jia Fan, Caicun Zhou

**Affiliations:** 1 Department of Medical Oncology, Shanghai Pulmonary Hospital, Tongji University, Shanghai, 200433 China; 2 Department of Oncology, The First Affiliated Hospital of Guangdong Pharmaceutical University, Guangzhou, China; 3 Department of Respiration, Shanghai Chest Hospital, Shanghai, China; 4 Oncology Chemotherapy Department, The First Affiliated Hospital of USTC (Anhui Provincial Hospital), Hefei, China; 5 Department of Thoracic Medical Oncology, Beijing Cancer Hospital, Beijing, China; 6 Department of Chemotherapy, Qilu Hospital of Shandong University, Jinan, China; 7 Department of Thoracic Tumor, Hubei Cancer Hospital, Wuhan, China; 8 Department of Medical Oncology, Zhongshan Hospital, Fudan University, Shanghai, China; 9 Respiratory and Critical Care Medicine, West China School of Medicine/West China Hospital of Sichuan University, Chengdu, China; 10 Department of Medical Oncology, Jilin Cancer Hospital, Changchun, China; 11 Department of Medical Oncology, Jiangsu Cancer Hospital, Jiangsu Institute of Cancer Research, The Affiliated Cancer Hospital of Nanjing Medical University, Nanjing, China; 12 Department of Medical Oncology, Zhengzhou Central Hospital, Zhengzhou, China; 13 Department of Interventional Radiology, Hunan Cancer Hospital, Changsha, China; 14 Department of Medical Oncology, The First Affiliated Hospital of Henan University of Science and Technology, Luoyang, China; 15 Thoracic Medicine Department, Hunan Cancer Hospital and The Affiliated Cancer Hospital of Xiangya School of Medicine, Central South University, Changsha, China; 16 Department of Gastroenterology, The Affiliated Cancer Hospital of Zhengzhou University, Henan Cancer Hospital, Zhengzhou, China; 17 Department of Oncology, Shengjing Hospital of China Medical University, Shenyang, China; 18 Clinical Research and Development, Jiangsu Hengrui Pharmaceuticals Co., Ltd, Shanghai, China; 19 Department of Liver Surgery and Transplantation, Zhongshan Hospital, Fudan University, Shanghai, 200032 China

**Keywords:** NSCLC, PD-1, Camrelizumab, Tyrosine kinase inhibitors, Famitinib

## Abstract

**Background:**

The combination of immune checkpoint inhibitors and antiangiogenic agents has been effective in treating multiple cancers. This was further explored in an open-label, multicenter phase 2 basket study (NCT04346381), which evaluated the antitumor activity and safety of camrelizumab (an anti-PD-1 antibody) plus famitinib (a receptor tyrosine kinase inhibitor) in patients with advanced solid tumors. We herein report the findings from the cohort of advanced NSCLC patients who progressed after treatment with platinum-doublet chemotherapy and immunotherapy.

**Methods:**

Eligible patients were enrolled and treated with camrelizumab (200 mg once every 3 weeks via intravenous infusion) and oral famitinib (20 mg once daily). The primary endpoint was the objective response rate (ORR). Secondary endpoints included the disease control rate (DCR), duration of response (DoR), progression-free survival (PFS), overall survival (OS), and safety.

**Results:**

Forty patients were enrolled in this cohort, with a median follow-up duration of 11.5 months. Three patients (7.5%) achieved a partial response, and 29 patients (72.5%) achieved stable disease. The ORR and DCR with this combination regimen were 7.5% (95% CI, 1.6–20.4) and 80.0% (95% CI, 64.4–90.9), respectively. The median DoR was 12.1 months (95% CI, 10.3-not reached). The median PFS was 5.4 months (95% CI, 4.1–7.5), and the median OS was 12.1 months (95% CI, 9.1–16.7). The estimated 12-month OS rate was 51.5% (95% CI, 34.9–65.9). The most frequent grade 3 or higher treatment-related adverse events occurring in more than 5% of patients included hypertension (27.5%), palmar-plantar erythrodysesthesia syndrome (10%), decreased neutrophil count (10%), and proteinuria (7.5%).

**Conclusion:**

Camrelizumab plus famitinib demonstrated favorable benefits in PFS and OS, along with manageable safety profiles, in patients with advanced NSCLC who progressed after platinum-doublet chemotherapy and immunotherapy. This finding warrants further exploration.

**Supplementary Information:**

The online version contains supplementary material available at 10.1007/s00262-024-03715-4.

## Introduction

The emergence of checkpoint inhibitors (ICIs), specifically monoclonal antibodies targeting programmed cell death 1 (PD-1) and its ligand (PD-L1), has revolutionized the therapeutic landscape for advanced non-small cell lung cancer (NSCLC) in cases without targetable driver mutations [1]. Although first-line ICIs, either alone or in combination with chemotherapy, have achieved extraordinary results in treating this patient population, the majority of them ultimately develop resistance to immunotherapy. However, even though the phase 3 REVEL [2] and LUME-Lung 1 trials [3] established the role of ramucirumab plus docetaxel (for all histology) and nintedanib in combination with docetaxel (only for adenocarcinoma histology) as second-line treatments in advanced NSCLC, the benefits of these regimens were marginal, with a median overall survival (OS) prolongation of about 1.4 months. Currently, single-agent docetaxel remains the standard option for patients with advanced NSCLC who progress after first-line platinum-doublet chemotherapy [4–7], yet it yields unsatisfactory clinical outcomes, with an objective response rate (ORR) of approximately 10% and a median OS of about 10 months. Therefore, novel and effective treatment options for pretreated NSCLC patients remain an unmet need in this setting.

Previous studies have shown that antiangiogenic agents can alleviate hypoxia in the tumor microenvironment (TME), promote the infiltration of immune CD8 + T cells, and enhance the efficacy of immunotherapy [8–10]. Lung-MAP S1800A, a phase 2 randomized trial, has reported that the combination of pembrolizumab and ramucirumab significantly prolonged OS compared to the docetaxel plus ramucirumab regimen [11]. In addition, the CONTACT 01 phase 3 trial investigated the efficacy and safety of atezolizumab combined with cabozantinib, compared to docetaxel monotherapy, in patients with metastatic NSCLC who had previously been treated with checkpoint inhibitors and chemotherapy. The trial observed a trend toward OS prolongation with a hazard ratio (HR) of 0.88, indicating the potential of antiangiogenesis to overcome resistance to immunotherapy [12]. Furthermore, another ongoing phase 3 trial (LEAP-008, NCT03976375) is evaluating the efficacy and safety of pembrolizumab plus lenvatinib compared to docetaxel in the treatment of NSCLC previously treated with chemo-immunotherapy [13].

The anti-PD-1 antibody camrelizumab, in combination with chemotherapy, has become the standard first-line regimen in China for patients with advanced NSCLC without *EGFR* or *ALK* genomic tumor aberrations [14, 15]. Famitinib (SHR1210), a novel and promising multitargeted tyrosine kinase inhibitor (TKI), inhibits the activity of c-kit, VEGFR-2, and PDGFRβ [16]. Previously, the combination of camrelizumab and famitinib has shown potent antitumor activity and tolerability in patients with advanced gynecologic and genitourinary malignancies [17–19]. Therefore, a basket phase 2 trial was initiated to further evaluate the efficacy and safety of the combination of camrelizumab and famitinib in various solid tumors. In this study, we report the results from cohort 7, which consisted of patients with advanced NSCLC who progressed after treatment with platinum-doublet chemotherapy and immunotherapy.

## Materials and methods

### Study design and patients

This is an open-label, multicentre, phase 2 basket study (NCT04346381, registered April 15, 2020) of camrelizumab plus famitinib for the treatment of patients with advanced solid tumors. Here, we present the data from cohort 7, consisting of patients with advanced NSCLC, previously treated with chemo-immunotherapy, regardless of histology type (squamous or non-squamous). Eligible patients had recurrent or metastatic NSCLC, confirmed by pathology or cytology, with wild-type *EGFR* and *ALK*. They were previously treated with platinum-doublet chemotherapy and anti-PD-1 or anti-PD-L1 agents (either combined or sequentially) and had progressed after no more than two lines of systemic therapy. Patients also met the following inclusion criteria: (1) age between 18 and 75 years; (2) Eastern Cooperative Oncology Group (ECOG) performance status of 0 or 1; (3) at least one measurable lesion as defined by the Response Evaluation Criteria in Solid Tumors (RECIST) v1.1; (4) a life expectancy of no less than 12 weeks, and 5) adequate organ function. The key exclusion criteria for this cohort were as follows: (1) any active autoimmune diseases or a history of such diseases; (2) concurrent use of immune-suppressants or systemic corticosteroids for immunosuppression within 2 weeks prior to the first dose of the study treatment; (3) poorly controlled hypertension; (4) untreated central nervous system metastases; (5) clinically significant cardiovascular disease; (6) concurrent treatment with anticoagulation therapies or a tendency for bleeding; (7) radiographic imaging indicating tumor invasion of a major blood vessel, unclear boundary with a blood vessel, or cavitation in lung lesions that might cause fatal bleeding, as determined by the investigator; (8) history of pulmonary embolism, stroke, or deep venous thrombosis within 6 months prior to the first dose of the study treatment; (9) active infection; or (10) diagnosis of other malignancies within the past 5 years.

This trial was conducted in strict adherence to the Declaration of Helsinki, Good Clinical Practice guidelines, and applicable local laws and regulatory requirements. The study protocol, along with any amendments, received approval from the independent Institutional Review Board or ethics committee at each participating study site. All participating patients provided their written informed consent. This trial was registered with ClinicalTrials.gov, number NCT04346381.

### Procedures

All patients enrolled in cohort 7 received a fixed dose of camrelizumab 200 mg via intravenous infusion over 30–60 min once every 3 weeks (Q3W) on Day 1 of each 3-week treatment cycle, and oral famitinib, at a starting dose of 20 mg once daily (QD), before or after a meal. Study treatment was continued until the occurrence of any of the following: intolerable toxicity, confirmed disease progression as per RECIST v1.1, decision by the investigator or patient, withdrawal of consent, poor adherence, or loss to follow-up, whichever came first. Patients with initial documented evidence of disease progression based on RECIST v1.1 could continue receiving study treatment if the investigator assessed the treatment as tolerable and providing clinical benefit. The study treatment was to continue until the next radiological confirmation of disease progression at least 4 weeks later. For camrelizumab, dose modifications were not permitted; however, dose interruptions of up to 12 weeks were allowed. The total duration of camrelizumab exposure should not exceed 24 months. Dose modifications of famitinib were permitted, including dose interruption, dose reduction (from the starting dose of 20 mg once daily to 15 mg once daily), and alteration in the dosing schedule to a 14 days on-7 days off regimen. However, once reduced or altered, the famitinib dose or dosing frequency could not be resumed to the original schedule.

### Endpoints

The primary endpoint was the ORR, as assessed by the investigator according to RECIST v1.1. ORR was defined as the proportion of patients whose best overall response (BOR) included either a confirmed complete response (CR) or a partial response (PR). Secondary endpoints included disease control rate (DCR), duration of response (DoR), time to treatment response (TTR), progression-free survival (PFS), OS, 6-, 9-, and 12-month OS rates, as well as the safety profile. DCR was defined as the proportion of patients whose BOR was either a CR, PR, or durable stable disease (SD). DoR was defined as the time from the first documented objective response until the first radiographic documentation of disease progression or death from any cause, whichever occurred first. PFS was defined as the time from the first dose of study treatment until the first documented disease progression per RECIST v1.1 or death from any cause, whichever occurred first. OS was defined as the time from the first dose of the study treatment until death from any cause. Exploratory endpoints involved exploring the correlations between efficacy (ORR and PFS) and potential biomarkers, such as PD-L1 tumor proportion score (TPS) and total tumor mutational burden (tTMB).

### Assessments

Tumor response assessments, per RECIST v1.1, were initially conducted at baseline and then every 9 weeks, as determined by the investigator's discretion. The first documentation of a CR or PR needed to be confirmed at least 4 weeks later. Additionally, an imaging examination was required for the confirmation of disease progression ≥ 4 weeks after the initial documented radiographic disease progression per RECIST v1.1, except in cases of rapid radiological or clinical progression. For patients who discontinued study treatment without radiological evidence of disease progression, tumor response assessment was performed every 3 months until documented disease progression, initiation of first subsequent anti-cancer therapy, loss to follow-up, death, or study termination by the sponsor, whichever occurred first. The survival status of patients was monitored and collected through clinical or remote follow-ups every 2 months until death. Safety evaluations included the monitoring of adverse events (AEs), measurement of vital signs, clinical laboratory tests, and 12-lead electrocardiogram assessments. AEs were recorded and monitored until at least 30 days after the last dose of study treatment. Patients were monitored for serious AEs and immune-mediated AEs until 90 days after the last dose of camrelizumab. The severity of AEs was assessed and graded based on the NCI Common Terminology Criteria for Adverse Events, version 5.0.

PD-L1 expression was obtained from some patients in clinical practice and measured using the IHC 22C3 pharmDx kit (Agilent Technologies, Santa Clara, CA). PD-L1 positivity was defined as a TPS of 1% or higher. TMB measurement in pretreatment tumor biopsy or archival tissue was typically conducted using the BGI Oseq pancancer panel, which covers 636 genes and 1.95 Mb, on the MGISEQ-2000 platform developed by MGI Tech Co. Ltd. (a subsidiary of the BGI Group). TMB was defined as the total number of somatic, coding, base substitutions, and indel mutations per megabase of the genome examined, and it was classified into TMB-high (≥ 10 mutations/Mb) and TMB-low (< 10 mutations/Mb) categories [20, 21].

### Statistical analyses

In the cohort 7, the desirable target ORR was set at 25%, while the uninteresting level of ORR under the null hypothesis was set at 10%. Based on these assumptions, with a one-sided α-error of 5% and a power of 70%, cohort 7 required the treatment of 19 evaluable patients during stage I. Stage II would proceed if at least 2 responders were obtained during stage I, subsequently involving the treatment of an additional 8 evaluable patients. Overall, the combination regimen would be considered active if 6 or more responders were observed among the 27 patients. In cohort 7, 2 out of the 19 patients enrolled in stage I achieved objective responses, and the actual sample size for the stage 2 was 21 patients. Overall, if a total of 9 or more responders were observed during the entire study period, the combination regimen in cohort 7 would be considered active. This critical value was estimated using the method described by Koyama and Chen [22].

Efficacy analyses were performed in all enrolled patients who had received at least one dose of study treatment, defined as the full analysis set (FAS). Safety was assessed in all patients who had received at least one study treatment and had at least one post-baseline safety evaluation. ORR and DCR, along with their 95% confidence intervals (CIs), were calculated using the Clopper-Pearson exact method based on the binomial distribution. The medians of time-to-event endpoints (DoR, PFS, and OS) were calculated using the Kaplan–Meier method, with their 95% CIs estimated using the Brookmeyer-Crowley method. The Kaplan–Meier method was used to estimate the OS probabilities at 6, 9, and 12 months. The corresponding 95% CIs were calculated using the log–log transformation (based on the normal approximation method) with back transformation to a CI on the untransformed scale. TTR was summarized using median, minimum and maximum. All statistical analyses were conducted using SAS, version 9.4.

## Results

### Patients

Between September 4, 2020, and July 12, 2021, a total of 40 patients with advanced NSCLC, who had previously been treated with platinum-doublet chemotherapy and ICIs (anti-PD-1 or anti-PD-L1) and progressed after ≤ 2 lines of systemic therapy, were enrolled in cohort 7 from 12 study centers in China. All patients enrolled in this cohort were treated with the combination regimen of camrelizumab plus famitinib and included in both the full analysis set and the safety analysis set.

The baseline demographics and clinical characteristics of patients are shown in Table 1. The median age of the patients was 62 years (range, 41–73), 90% (36/40) of whom were male, and 67.5% (27/40) were current or former smokers. The majority of patients had an ECOG PS of 1 (36/40; 90%). Regarding histology, 23 (57.5%) patients had non-squamous carcinomas, and 17 (42.5%) had squamous carcinomas. Thirty-nine (97.5%) patients had metastases irrespective of the organs involved, with brain metastasis observed in 2 (5.0%) and liver metastases in 3 (7.5%) patients. Additionally, 17 patients (42.5%) had received two prior lines of systemic therapy.

**Table 1:** Demographics and baseline characteristics

	All patients (n = 40)
Age, years, median (range)	62 (41–73)
< 65	27 (67.5)
≥ 65	13 (32.5)
Sex	
Male	36 (90.0)
Female	4 (10.0)
ECOG performance status	
0	4 (10.0)
1	36 (90.0)
Smoking status^a^	
Never	9 (22.5)
Current	2 (5.0)
Former	25 (62.5)
Histologic type	
Squamous carcinoma	17 (42.5)
Non-squamous carcinoma	23 (57.5)
Presence of metastases	39 (97.5)
Brain metastases	
Yes	2 (5.0)
No	38 (95.0)
Liver metastases	
Yes	3 (7.5)
No	37 (92.5)
No. of prior systemic therapy	
1	23 (57.5)
2	17 (42.5)
PD-L1 TPS^b^	
< 1%	27 (67.5)
≥ 1%	11 (27.5)
1–49%	6 (15.0)
≥ 50%	5 (12.5)
tTMB^b^	
Low (< 10 mutations/Mb)	7 (17.5)
High (≥ 10 mutations/Mb)	17 (42.5)

Data are n (%) unless otherwise indicated

ECOG, Eastern Cooperative Oncology Group; PD-L1, programmed death-ligand 1; TPS, tumor proportion score; tTMB, tissue tumor mutational burden

^a^Data were missing for the remaining patients

^b^A mandatory fresh biopsy or archival tissue for PD-L1 TPS or tTMB was not requested at enrollment

**Fig. 1:**
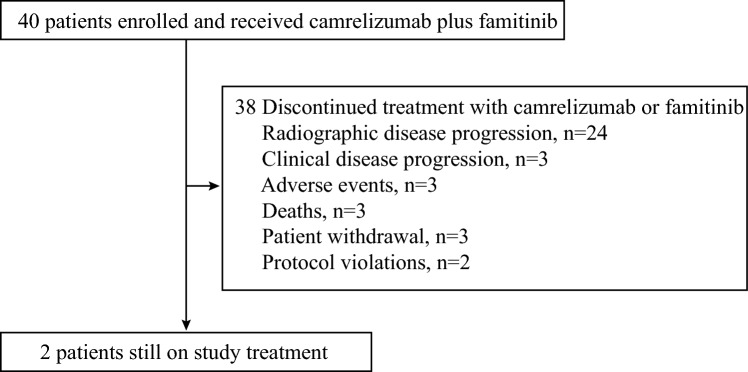
Study flow diagram of cohort 7

According to laboratory assessment, tumor PD-L1 expression data were available for 38 patients; of these, 11 (27.5%) patients had a PD-L1 TPS of ≥ 1%, and 5 (12.5%) patients had a PD-L1 TPS of ≥ 50%. As of the cutoff date (June 22, 2022), the median follow-up duration was 11.5 months (range, 1.7–18.1). Among the 40 patients, 2 (5.0%) were still receiving study treatment at the time of analysis. The reasons for discontinuing treatment with camrelizumab or famitinib included disease progression (either radiographic or clinic) in 27 (67.5%) patients, AEs in 3 (7.5%) patients, death in 3 (7.5%) patients, patient withdrawal in 3 (7.5%) patients, and protocol violations in 2 (5.0%) patients (Fig. 1).

### Anti-tumor activity in all patients

**Fig. 2:**
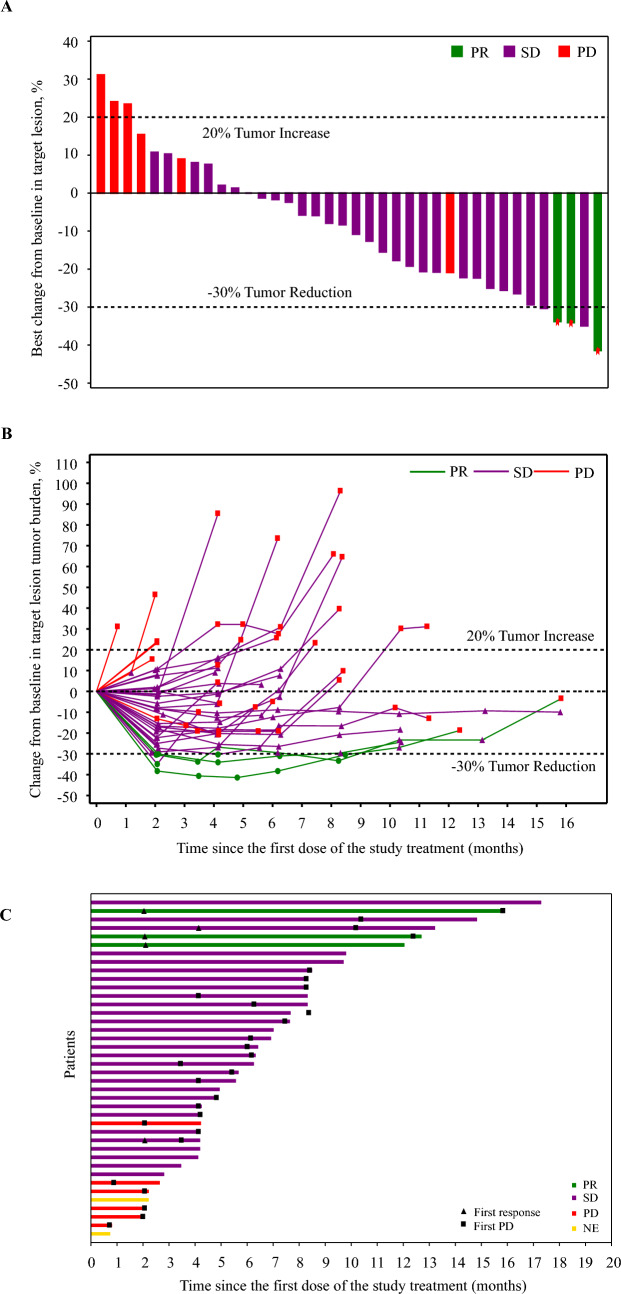
Antitumor activity of camrelizumab plus famitinib in cohort 7 patients. **A** Best percentage change from baseline in the size of target lesions. Red asterisks indicated confirmed responses. **B** Percentage change in the tumor burden of the target lesion from baseline over time. **C** Treatment response and duration of response

Among the 40 patients in the FAS, 3 (7.5%) achieved a PR, and 29 (72.5%) achieved SD as their best response. The confirmed ORR and DCR with this combination regimen were 7.5% (95% CI, 1.6–20.4) and 80.0% (95% CI, 64.4–90.9), respectively (Table 2). Overall, the best change in the sum of diameter of target lesion from baseline for each patient is plotted in Fig. 2A, with a decrease in tumor burden observed in 26 (65.0%) patients. Tumor responses since the initial dose of the study treatment, as shown in Fig. 2B, indicated that reductions in tumor burden were observed over several assessments. The median TTR was 2.1 months (range, 2.0–2.1), and the median DoR was 12.1 months (95% CI, 10.3-not reached [NR]; Fig. 2C).

**Table 2:** Summary of tumor responses and survival data

	All patients (n = 40)
Best overall response, n (%)	
Partial response	3 (7.5)
Stable disease	29 (72.5)
Progressive disease	6 (15.0)
Not evaluable	2 (5.0)
ORR, % (95% CI)	7.5 (1.6–20.4)
DCR, % (95% CI)	80.0 (64.4–90.9)
Time to response, months, median (range)	2.1 (2.0–2.1)
Duration of response, months, median (95% CI)	12.1 (10.3–NR)
Progression-free survival, months, median (95% CI)	5.4 (4.1–7.5)
Overall survival, months, median (95% CI)	12.1 (9.1–16.7)
6-month rate, % (95% CI)	80.0 (64.0–89.5)
9-month rate, % (95% CI)	67.5 (50.7–79.7)
12-month rate, % (95% CI)	51.5 (34.9–65.9)

ORR, objective response rate; CI, confidence interval; DCR, disease control rate; NR, not reached

**Fig. 3:**
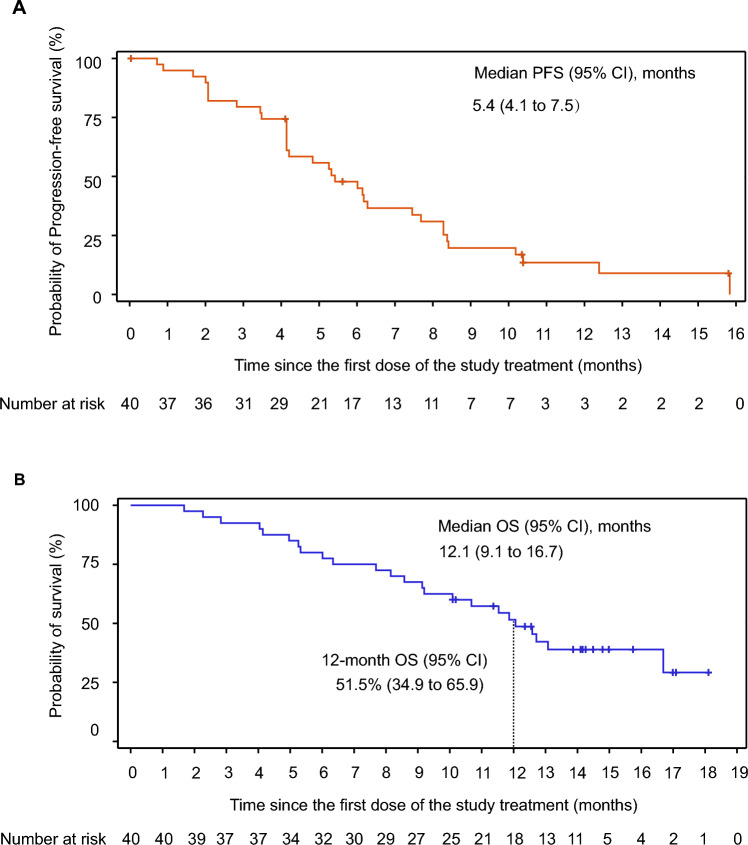
Kaplan–Meier estimates of PFS **A** and OS **B** in cohort 7 patients. PFS, progression-free survival; OS, overall survival; CI, confidence interval

At the data cutoff, 34 (85.0%) patients experienced events of investigator-assessed disease progression or death. The median PFS, estimated using the Kaplan–Meier method, was 5.4 months (95% CI, 4.1–7.5; Fig. 3A). The 6-, 9-, and 12-month PFS rates were 47.8% (95%CI, 31.4–62.5), 19.7% (95% CI, 8.7–33.8), and 13.5% (95% CI, 4.7–26.9), respectively. In cohort 7, a total of 24 patients (60.0%) had died. The median OS was 12.1 months (95% CI, 9.1–16.7; Fig. 3B), with the Kaplan–Meier estimated 6-, 9-, and 12-month OS rates being 80.0% (95% CI, 64.0–89.5), 67.5% (95% CI, 50.7–79.7), and 51.5% (95% CI, 34.9–65.9), respectively.

### Anti-tumor activity by PD-L1 TPS or tTMB status

Anti-tumor activity outcomes by PD-L1 TPS or tTMB are shown in the Table S1. Among the 40 patients, 38 (95.0%) had available data on tumor PD-L1 expression. The ORR was 7.4% (95% CI, 0.9–24.3) in 27 patients with PD-L1 TPS < 1% vs. 9.1% (95% CI, 0.2–41.3) in 11 patients with PD-L1 TPS ≥ 1%. The median PFS was 5.3 months (95% CI, 4.1–8.3) for patients with PD-L1 TPS < 1%, and 6.1 months (95% CI, 1.7-NR) for those with PD-L1 TPS ≥ 1%. TMB data were available for 24 (60.0%) of the patients, among whom 7 had low tTMB (< 10 mutations/Mb) and 17 had high tTMB (≥ 10 mutations/Mb). Subgroup analysis revealed an ORR of 0% (95% CI, 0.0–41.0) in patients with low tTMB compared to 11.8% (95% CI, 1.5–36.4) in those with high tTMB. The median PFS was 5.3 months (95% CI, 0.7–7.7) for patients with low tTMB and 6.2 months (95% CI, 3.5–12.4) for those with high tTMB.

### Safety

The median duration of exposure to camrelizumab was 5.9 months (range, 0.7–17.2), with a relative dose intensity of 99.6% (range, 66.7–108.6). The median duration of exposure to famitinib was 5.4 months (range, 0.4–17.0), with a median relative dose intensity of 82.4% (range, 52.0–100.0).

Thirty-eight patients experienced one or more TRAEs of any grade (Table 3), with the most frequent TRAEs being decreased platelet count (n = 24, 60.0%), hypertension (n = 23, 57.5%), proteinuria (n = 22, 55.0%), and decreased neutrophil count (n = 21, 52.5%). Twenty-six (60.0%) patients had grade 3 or higher TRAEs, with the most frequent occurring in more than 5% of patients being hypertension (n = 11, 27.5%), palmar-plantar erythrodysesthesia syndrome (n = 4, 10%), decreased neutrophil count (n = 4, 10%), and proteinuria (n = 3, 7.5%). Reactive cutaneous capillary endothelial proliferation (RCCEP), a clinically manageable and self-resolving TRAE associated with camrelizumab monotherapy, occurred in only one patient (2.5%, grade 1) of this cohort treated with the combination of camrelizumab and famitinib.

**Table 3:** Treatment-related adverse events occurring in at least 10% of patients

	All patients (n = 40)	
	Any grade	Grade ≥ 3
Any TRAEs	38 (95.0)	24 (60.0)
Platelet count decreased	24 (60.0)	2 (5.0)
Hypertension	23 (57.5)	11 (27.5)
Proteinuria	22 (55.0)	3 (7.5)
Neutrophil count decreased	21 (52.5)	4 (10.0)
Anemia	20 (50.0)	1 (2.5)
White blood cell count decreased	18 (45.0)	2 (5.0)
Occult blood positive	18 (45.0)	0
PPE syndrome	17 (42.5)	4 (10.0)
Aspartate aminotransferase increased	14 (35.0)	1 (2.5)
Alanine aminotransferase increased	11 (27.5)	2 (5.0)
Diarrhea	11 (27.5)	0
Lymphocyte count decreased	6 (15.0)	0
Hypertriglyceridemia	5 (12.5)	2 (5.0)
Asthenia	5 (12.5)	0
Decreased appetite	5 (12.5)	0
Blood bilirubin increased	5 (12.5)	0
Hypothyroidism	5 (12.5)	0
Hematuria	5 (12.5)	0
Weight decreased	5 (12.5)	0
Hyperglycemia	5 (12.5)	0
Blood creatinine increased	5 (12.5)	0
Blood lactate dehydrogenase increased	5 (12.5)	0
Hyperuricemia	5 (12.5)	0
Hypokalemia	4 (10.0)	1 (2.5)
Lipase increased	4 (10.0)	1 (2.5)
Rash	4 (10.0)	0
Blood alkaline phosphatase increased	4 (10.0)	0
Hyperlipidemia	4 (10.0)	0
Urinary occult blood positive	4 (10.0)	0
Hyponatremia	4 (10.0)	0

Data are shown in n (%)

TRAE, treatment-related adverse event; PPE, palmar-plantar erythrodysesthesia

In cohort 7, serious TRAEs of grade 3 or higher occurred in 9 (22.5%) patients, including increased alanine aminotransferase, hemoptysis, sudden death, acute kidney injury, renal impairment, herpes zoster, urinary tract infection, pneumonia fungal, drug-induced liver injury, cerebral infarction, cerebral hemorrhage, arthralgia, hyperthyroidism, and hypertension (n = 1 for each; 2.4%) (Table S2. Four (10.0%) deaths were considered as treatment-related, including one sudden death, one hemoptysis, one from cerebral infarction and cerebral hemorrhage, and one from acute kidney injury and drug-induced liver injury.

Three (7.5%) patients discontinued camrelizumab due to TRAEs, which included cerebral hemorrhage, cerebral infarction, acute kidney injury, urinary tract infection, renal impairment, and drug-induced liver injury (n = 1 for each, 2.5%). Three (7.5%) patients discontinued famitinib because of TRAEs that were cerebral infarction, cerebral hemorrhage, acute kidney injury, renal impairment, and drug-induced liver injury (n = 1 for each, 2.5%). TRAEs leading to dose interruption or dose modification (of famitinib) are shown in the Table S3. Eleven (27.5%) patients experienced at least one TRAE leading to camrelizumab interruption, with increased alanine aminotransferase (n = 3, 7.5%) and increased aspartate aminotransferase (n = 2, 5.0%) occurring in more than one patient. Dose interruptions of famitinib due to TRAEs occurred in 26 (65.0%) patients, with proteinuria (n = 6, 15.0%), decreased platelet count (n = 6, 15.0%), decreased white blood cell count (n = 5, 12.5%), hypertension (n = 4, 10.0%), and decreased neutrophil count (n = 3, 7.5%) occurring in more than 2 patients. TRAEs leading to dose reduction of famitinib were reported in 19 (47.5%) patients, with palmar-plantar erythrodysesthesia (PPE) syndrome (n = 4, 10.0%), decreased platelet count decreased (n = 3, 7.5%), proteinuria (n = 3, 7.5%), and decreased neutrophil count (n = 3, 7.5%) occurring in more than 2 patients. Five (12.5%) patients experienced at least one TRAE leading to a reduction in the dose frequency of famitinib, including increased alanine aminotransferase, hypercholesterolemia, hypertriglyceridemia, PPE syndrome, decreased white blood cell count, and proteinuria (n = 1 for each, 2.5%).

Immune-mediated AEs of any grade, regardless of attribution to study treatment as assessed by investigators, were observed in 5 (12.5%) patients (Table S4). These AEs included hypothyroidism (n = 2, 5.0%), hyperthyroidism, drug-induced liver injury, increased alpha hydroxybutyrate dehydrogenase, increased blood creatine phosphokinase, increased blood lactate dehydrogenase, and interstitial lung disease (n = 1 for each, 2.5%).

## Discussion

We report for the first time on the antitumor activity and safety profile of the combination of camrelizumab and famitinib in patients with advanced NSCLC, who had progressed after treatment with platinum-doublet chemotherapy and immunotherapy. Although the primary endpoint of antitumor activity, indicated by an ORR of 7.5%, was not met, this combination regimen demonstrated meaningful clinical benefits in secondary endpoints, including PFS and OS. These findings suggest the potential efficacy of camrelizumab and famitinib for this specific group of patients with advanced NSCLC.

In this cohort, the median PFS and OS were 5.4 months and 12.1 months, respectively, with a 12-month OS rate of 51.5%. Interestingly, despite potential issues in cross-trial comparisons due to heterogeneous populations and varying study designs, the median PFS observed with camrelizumab plus famitinib was numerically longer than that achieved with docetaxel alone (2.8–4.2 months) [2, 4–7], and ramucirumab plus docetaxel (4.5 months) [2], in similar clinical settings. Moreover, the median OS in this cohort appeared to be more favorable than that achieved with docetaxel alone (6.0–9.6 months) [2, 4–7], and was comparable to the OS observed with ramucirumab plus docetaxel (10.5 months) [2]. Notably, a considerable proportion of patients enrolled in this cohort exhibited fragility due to heavy pretreatment, with 42.5% having received two prior lines of systemic therapy and receiving this combination regimen as third-line therapy. Therefore, our findings suggest that this combination regimen can provide an impressive survival benefit (both PFS and OS) in this patient population.

PD-L1 expression and TMB have emerged as promising biomarkers for response to ICI monotherapy in NSCLC [14, 20, 23–25]. In this cohort, we observed a numerically higher ORR (9.1% vs. 7.4%) and a slightly prolonged median PFS (6.1 vs. 5.3 months) in patients with a PD-L1 TPS of ≥ 1% compared to those with a PD-L1 TPS of < 1%. Furthermore, patients with high tTMB (≥ 10 mutations/Mb) achieved a better ORR (11.8% vs. 0%) and longer median PFS (6.2 vs. 5.3 months) than those with low tTMB (< 10 mutations/Mb). These findings suggest that adding antiangiogenic TKIs to anti-PD-1 immunotherapy may restore ICI sensitivity in patients of this cohort, particularly for those with a PD-L1 TPS of ≥ 1% or high genome instability. However, the interpretation of the correlation between these biomarkers and efficacy should be approached with caution due to the exploratory nature of the analyses and the limited number of patients in the subgroups, especially when compared to similar studies.

In this cohort, the overall incidence rate of grade 3 or higher TRAEs with camrelizumab plus famitinib was comparable to those reported in trials of advanced or metastatic urothelial carcinoma, ovarian cancer, or renal cell carcinoma [17–19], and no new safety signals were detected. The most common grade 3 or higher TRAEs observed with this combination treatment included hypertension, proteinuria, and PPE syndrome. These TRAEs might be related to famitinib [26–28] and are representative of the anti-angiogenic effects of VEGF/VEGFR TKIs [29, 30]. Meanwhile, RCCEP, a common skin adverse reaction caused by camrelizumab monotherapy, occurred in approximately 67–97% of patients with various advanced solid tumors [31]. However, in this cohort, the incidence of RCCEP with the combination of camrelizumab and famitinib was only 2.5%, significantly lower than that reported with camrelizumab monotherapy. These results aligned with previous findings involving camrelizumab combined with VEGFR TKIs [17–19, 32–34]. As a result of this combination regimen, there was a slight increase in the incidence of TRAEs leading to a dose reduction of famitinib (n = 19, 47.5%; of these, 5 patients experienced TRAEs necessitating modification in the famitinib dose frequency). Despite this, the rate of discontinuation due to TRAEs was low (5.0% for camrelizumab and 7.5% for famitinib), which might contribute to the favorable PFS and OS benefits observed in this study.

The current study has several potential limitations. First, the trial was constrained by its exploratory nature as a single-arm phase 2 design, lacking a control arm of either docetaxel or ICI monotherapy. Consequently, further prospective randomized controlled trials are necessary. Second, there is a potential for inherent bias based on the investigator’s assessment of the ORR and PFS. Third, some patients had non-evaluable samples for PD-L1 or tTMB analysis, as providing these samples was not a mandatory requirement for enrollment. Therefore, identifying predictive biomarkers to select suitable candidates for the combination of antiangiogenic agents and immunotherapy in this clinical setting still needs to be addressed.

## Conclusions

The combination of camrelizumab and famitinib resulted in acceptable PFS and OS benefits which warrants further investigations; safety issues emerged in this cohort highlighting the need to explore predictive factors for serious AEs when combining antiangiogenic agents and ICIs in pretreated NSCLC patients.

### Supplementary Information

Below is the link to the electronic supplementary material.

Supplementary file1 (DOCX 26 kb)

## Data Availability

The datasets used and/or analyzed during the current study are available from the corresponding author on reasonable request.

## Abbreviations

AE: Adverse event
CI: Confidence interval
CR: Complete response
DCR: Disease control rate
DoR: Duration of response
ECOG: Eastern cooperative oncology group
EGFR: Epidermal growth factor receptor
FAS: Full analysis set
HR: Hazard ratio
ICI: Immune checkpoint inhibitor
NSCLC: Non-small cell lung cancer
NR: Not reached
ORR: Objective response rate
OS: Overall survival
PD-1: Programmed cell death protein 1
PD-L1: Programmed death-ligand 1
PFS: Progression-free survival
PPE: Palmar-plantar erythrodysesthesia syndrome
PR: Partial response
Q3W: Once every 3 weeks
QD: Once daily
RCCEP: Reactive cutaneous capillary endothelial proliferation
RECIST: Response evaluation criteria in solid tumors
SD: Stable disease
tTMB: Total tumor mutational burden
TKI: Tyrosine kinase inhibitor
TME: Tumor microenvironment
TPS: Tumor proportion score
TRAE: Treatment-related adverse event
TTR: Time to treatment response
