# Supplementary material for: Camrelizumab plus famitinib in previously chemo-immunotherapy treated patients with advanced NSCLC: results from an open-label multicenter phase 2 basket study
Source: Cancer Immunol Immunother. 2024 May 10;73(7):124. doi: 10.1007/s00262-024-03715-4 (PMC11087418; doi:10.1007/s00262-024-03715-4)
Supplement: Supplementary file 1 — Supplementary file1 (DOCX 26 kb) [file 262_2024_3715_MOESM1_ESM.docx]

# Table S1. Summary of tumor responses assessed by the investigator and survival data in relation to PD-L1 TPS or tTMB status.

|  | **PD-L1 TPS <1%**  **(n = 27)** | **PD-L1 TPS ≥1%**  **(n = 11)** | **tTMB-Low**  **(<10 mutations/Mb)**  **(n = 7)** | **tTMB-High**  **(≥10 mutations/Mb)**  **(n = 17)** |
| --- | --- | --- | --- | --- |
| Best overall response, n (%) |  |  |  |  |
| Partial response | 2 (7.4) | 1 (9.1) | 0 | 2 (11.8) |
| Stable disease | 21 (77.8) | 7 (63.6) | 6 (85.7) | 13 (76.5) |
| Progressive disease | 4 (14.8) | 1 (9.1) | 1 (14.3) | 1 (5.9) |
| Not evaluable | 0 | 2 (18.2) | 0 | 1 (5.9) |
| ORR, % (95% CI) | 7.4 (0.9–24.3) | 9.1 (0.2–41.3) | 0.0 (0.0–41.0) | 11.8 (1.5–36.4) |
| DCR, % (95% CI) | 85.2 (66.3–95.8) | 72.7 (39.0–94.0) | 85.7 (42.1–99.6) | 88.2 (63.6–98.5) |
| PFS, months, median (95% CI) | 5.3 (4.1–8.3) | 6.1 (1.7–NR) | 5.3 (0.7–7.7) | 6.2 (3.5–12.4) |

PD-L1, programmed death-ligand 1; TPS, tumor proportion score; tTMB, tissue tumor mutational burden; ORR, objective response rate; CI, confidence interval; DCR, disease control rate; NR, not reached; PFS, progression-free survival.

# Table S2. Serious treatment-related adverse events.

|  | **All patients (n=40)** | |
| --- | --- | --- |
|  | **Any grade** | **Grade ≥3** |
| Any serious TRAEs | 12 (30.0) | 9 (22.5) |
| Alanine aminotransferase increased | 2 (5.0) | 1 (2.5) |
| Hemoptysis | 1 (2.5) | 1 (2.5) |
| Sudden death | 1 (2.5) | 1 (2.5) |
| Acute kidney injury | 1 (2.5) | 1 (2.5) |
| Renal impairment | 1 (2.5) | 1 (2.5) |
| Herpes zoster | 1 (2.5) | 1 (2.5) |
| Urinary tract infection | 1 (2.5) | 1 (2.5) |
| Pneumonia fungal | 1 (2.5) | 1 (2.5) |
| Drug-induced liver injury | 1 (2.5) | 1 (2.5) |
| Cerebral infarction | 1 (2.5) | 1 (2.5) |
| Cerebral hemorrhage | 1 (2.5) | 1 (2.5) |
| Arthralgia | 1 (2.5) | 1 (2.5) |
| Hyperthyroidism | 1 (2.5) | 1 (2.5) |
| Hypertension | 1 (2.5) | 1 (2.5) |
| Aspartate aminotransferase increased | 1 (2.5) | 0 |
| Troponin T increased | 1 (2.5) | 0 |
| Diarrhea | 1 (2.5) | 0 |
| Proteinuria | 1 (2.5) | 0 |
| Gastroenteritis | 1 (2.5) | 0 |
| Hypopituitarism | 1 (2.5) | 0 |

Data are shown in n (%).

TRAE, treatment-related adverse event.

# Table S3. Treatment-related adverse events leading to dose modification.

|  | **All patients (n=40)** | |
| --- | --- | --- |
|  | **Any grade** | **Grade ≥3** |
| **TRAEs leading to interruption of camrelizumab** | 11 (27.5) | 6 (15.0) |
| Alanine aminotransferase increased | 3 (7.5) | 2 (5.0) |
| Aspartate aminotransferase increased | 2 (5.0) | 1 (2.5) |
| Hypokalemia | 1 (2.5) | 1 (2.5) |
| Hypercholesterolemia | 1 (2.5) | 1 (2.5) |
| Hypertriglyceridemia | 1 (2.5) | 1 (2.5) |
| Urinary tract infection | 1 (2.5) | 1 (2.5) |
| Platelet count decreased | 1 (2.5) | 1 (2.5) |
| Pneumonia fungal | 1 (2.5) | 1 (2.5) |
| Hyponatremia | 1 (2.5) | 0 |
| Asthenia | 1 (2.5) | 0 |
| Pneumonitis | 1 (2.5) | 0 |
| Diarrhea | 1 (2.5) | 0 |
| Troponin T increased | 1 (2.5) | 0 |
| Hypothyroidism | 1 (2.5) | 0 |
| Interstitial lung disease | 1 (2.5) | 0 |
| Stomatitis | 1 (2.5) | 0 |
| Hematuria | 1 (2.5) | 0 |
| **TRAEs leading to interruption of famitinib** | 26 (65.0) | 14 (35.0) |
| Proteinuria | 6 (15.0) | 3(7.5) |
| Platelet count decreased | 6 (15.0) | 2 (5.0) |
| White blood cell count decreased | 5 (12.5) | 1 (2.5) |
| Hypertension | 4 (10.0) | 3 (7.5) |
| Neutrophil count decreased | 3 (7.5) | 2 (5.0) |
| Alanine aminotransferase increased | 2 (5.0) | 1 (2.5) |
| Aspartate aminotransferase increased | 2 (5.0) | 1 (2.5) |
| PPE syndrome | 2 (5.0) | 1 (2.5) |
| Diarrhea | 2 (5.0) | 0 |
| Troponin T increased | 2 (5.0) | 0 |
| Cerebral infarction | 1 (2.5) | 1 (2.5) |
| Herpes zoster | 1 (2.5) | 1 (2.5) |
| Hypertriglyceridemia | 1 (2.5) | 1 (2.5) |
| Urinary tract infection | 1 (2.5) | 1 (2.5) |
| Asthenia | 1 (2.5) | 0 |
| Non-infective gingivitis | 1 (2.5) | 0 |
| Mouth ulceration | 1 (2.5) | 0 |
| Stomatitis | 1 (2.5) | 0 |
| Oesophageal pain | 1 (2.5) | 0 |
| Gastrointestinal hemorrhage | 1 (2.5) | 0 |
| Gastroenteritis | 1 (2.5) | 0 |
| Blood creatinine increased | 1 (2.5) | 0 |
| Blood urea increased | 1 (2.5) | 0 |
| Hematuria | 1 (2.5) | 0 |
| **TRAEs leading to dose reduction of famitinib** | 19 (47.5) | 9 (22.5) |
| PPE syndrome | 4 (10.0) | 3 (7.5) |
| Neutrophil count decreased | 3 (7.5) | 1 (2.5) |
| Proteinuria | 3 (7.5) | 0 |
| Platelet count decreased | 3 (7.5) | 0 |
| Hypertension | 2 (5.0) | 2 (5.0) |
| Alanine aminotransferase increased | 2 (5.0) | 1 (2.5) |
| Arthralgia | 2 (5.0) | 1 (2.5) |
| Hyponatremia | 1 (2.5) | 1 (2.5) |
| Dysphonia | 1 (2.5) | 1 (2.5) |
| Hypercholesterolemia | 1 (2.5) | 1 (2.5) |
| Hypertriglyceridemia | 1 (2.5) | 1 (2.5) |
| Hyperthyroidism | 1 (2.5) | 1 (2.5) |
| White blood cell count decreased | 1 (2.5) | 0 |
| Glomerular filtration rate decreased | 1 (2.5) | 0 |
| Aspartate aminotransferase increased | 1 (2.5) | 0 |
| Taste disorder | 1 (2.5) | 0 |
| **TRAEs leading to modification in dose frequency of famitinib** | 5 (12.5) | 3 (7.5) |
| Alanine aminotransferase increased | 1 (2.5) | 1 (2.5) |
| Hypercholesterolemia | 1 (2.5) | 1 (2.5) |
| Hypertriglyceridemia | 1 (2.5) | 1 (2.5) |
| PPE syndrome | 1 (2.5) | 1 (2.5) |
| White blood cell count decreased | 1 (2.5) | 0 |
| Proteinuria | 1 (2.5) | 0 |
| Neutrophil count decreased | 1 (2.5) | 0 |

Data are shown in n (%).

TRAEs, treatment-related adverse events; PPE, palmar-plantar erythrodysesthesia.

# Table S4. Immune-mediated adverse events regardless of attribution to study treatment

|  | **All patients (n=40)** | |
| --- | --- | --- |
|  | **Any grade** | **Grade ≥3** |
| Any immune-mediated AEs | 5 (12.5) | 2 (5.0) |
| Hypothyroidism | 2 (5.0) | 0 |
| Hyperthyroidism | 1 (2.5) | 1 (2.5) |
| Drug-induced liver injury | 1 (2.5) | 1 (2.5) |
| Alpha hydroxybutyrate dehydrogenase increased | 1 (2.5) | 0 |
| Blood creatine phosphokinase increased | 1 (2.5) | 0 |
| Blood lactate dehydrogenase increased | 1 (2.5) | 0 |
| Interstitial lung disease | 1 (2.5) | 0 |

Data are shown in n (%).

AEs, adverse events.
